# A Study of the Protective Effect of Bushen Huoxue Prescription on Cerebral Microvascular Endothelia Based on Proteomics and Bioinformatics

**DOI:** 10.1155/2022/2545074

**Published:** 2022-01-06

**Authors:** Shao-Yang Zhao, Huan-Huan Zhao, Yi-Ming Li, Bao-Hua Wang, Sai-Mei Li

**Affiliations:** ^1^Department of Endocrinology, The First Affiliated Hospital of Guangzhou University of Chinese Medicine, Guangzhou 510405, Guangdong, China; ^2^The First School of Clinical Medicine, Guangzhou University of Chinese Medicine, Guangzhou 510405, Guangdong, China; ^3^Nutrition Department, Linyi People's Hospital, Linyi 276000, Shandong, China

## Abstract

Diabetic cognitive dysfunction is a serious complication of type 2 diabetes mellitus (T2DM), which can cause neurological and microvascular damage in the brain. At present, there is no effective treatment for this complication. Bushen Huoxue prescription (BSHX) is a newly formulated compound Chinese medicine containing 7 components. Previous research indicated that BSHX was neuroprotective against advanced glycosylation end product (AGE)-induced PC12 cell insult; however, the effect of BSHX on AGE-induced cerebral microvascular endothelia injury has not been studied. In the current research, we investigated the protective effects of BSHX on AGE-induced injury in bEnd.3 cells. Our findings revealed that BSHX could effectively protect bEnd.3 cells from apoptosis. Moreover, we analyzed the network regulation effect of BSHX on AGE-induced bEnd.3 cells injury at the proteomic level. The LC-MS/MS-based shotgun proteomics analysis showed BSHX negatively regulated multiple AGE-elicited proteins. Bioinformatics analysis revealed these differential proteins were involved in multiple processes, such as Foxo signaling pathway. Further molecular biology analysis confirmed that BSHX could downregulate the expression of FoxO1/3 protein and inhibit its nuclear transfer and inhibit the expression of downstream apoptotic protein Bim and the activation of caspase, so as to play a protective role in AGE-induced bEnd.3 injury. Taken together, these findings demonstrated the role of BSHX in the management of diabetic cerebral microangiopathy and provide some insights into the proteomics-guided pharmacological mechanism study of traditional Chinese Medicine.

## 1. Introduction

Diabetes mellitus (DM) is a common metabolic disease that can be accompanied by a variety of complications. Diabetic cognitive dysfunction is a major central nervous system complication of DM, mainly manifested as cognitive impairment and neurodegeneration [1–3]. Although the pathogenesis of this complication is still not clear, recent studies have found that overproduction of advanced glycosylation end products (AGEs) is closely related to the occurrence of cognitive dysfunction [4, 5]. AGEs are toxic substances produced by nonenzymatic glycosylation of proteins and reducing sugars under long-term hyperglycemia. The accumulation of AGEs in the brain can bring about progressive neurostructural changes and functional deficits through different pathways [6–9]. In addition, AGEs can damage cells and even kill them by inducing oxidative stress and reactive oxygen species (ROS) formation [4, 10, 11]. Cerebral microvascular endothelial cells are the basic oxygen supply unit of brains and the blood-brain barrier. In diabetic patients with long-term hyperglycemia, the intense nonenzymatic glycosylation reactions provoke the accumulation of AGEs in the vascular endothelium and thus result in vascular endothelial damage and dysfunction [12].

Bushen Huoxue prescription (BSHX) is a newly formulated compound Chinese medicine containing seven components, as listed in Table 1. Previous studies have established that its main components can effectively reduce blood glucose and improve cerebral microcirculatory disturbance and cognitive impairment [13–16]. Icariin (ICA) and Schizandrin A are the two bioactive ingredients isolated from BSHX. Our previous research results have shown that ICA could specifically bind to the target protein Bax to inhibit its migration to mitochondria, thereby reducing the production of endogenous ROS induced by AGEs and exerting an antiapoptotic effect [17]. In addition, Schizandrin A could alleviate neuron apoptosis and inflammation injury exposed to microglia-conditioned medium. The anti-inflammation mechanism of Schizandrin A includes the inhibition of NF-*κ*B phosphorylation and activation of MAPK pathway. What's more, BSHX has been proven effective clinically in improving diabetic dementia, but it lacks the support of systematic pharmacodynamic evaluation.

Proteomics is a systematic study of the expression, function, and interaction of all proteins in cells. The proteomics technology can reveal the entire protein change spectrum under specific physiological conditions and the effect of drugs on the changes of the whole proteome. Proteomics can be used to analyze the protein differences of diseases before and after drug administration, so as to discover and identify characteristic target proteins. Bioinformatics or systems biology methods can further identify the relevant signal transduction pathways, which can help us explain the pathogenesis of diseases and provide ideas for the diagnosis and treatment of diseases. In this study, the AGE-induced bEnd.3 cell injury model was used to study the protective effect of BSHX on cerebral microvascular endothelial cells. Combined with proteomics technology, the relevant signal pathways of BSHX inhibiting vascular endothelial injury were analyzed from the overall protein level, so as to reveal the molecular mechanism of BSHX in protecting cerebral microvascular endothelial cells.

## 2. Materials and Methods

### 2.1. Materials

BSHX granules were purchased from Beijing Kangrentang Pharmaceutical Co. Ltd. (Beijing, China). The medicinal components of BSHX are listed in Table 1. AGEs were purchased from Beijing Biosynthesis Biotechnology Co. Ltd. (Beijing, China) with a purity greater than 95% by high-performance liquid chromatography (HPLC). Antibodies against cleaved caspase-3, Bim, Histone H3, FoxO1, FoxO3a, *α*-Tubulin, and rabbit IgG were obtained from Cell Signaling Technology (Boston, MA, USA). 3-(4,5-Dimethylthiazol-2-yl)2,5-diphenyltetrazolium bromide (MTT) was bought from Sigma-Aldrich Chemical Co. (Saint Louis, MO, USA). The Dulbecco's modified eagle's medium (DMEM) and fetal bovine serum (FBS) were purchased from PAN-Biotech GmbH (Aidenbach, Germany). The endothelial cell medium (ECM) was obtained from Beijing M&C Gene Technology Ltd. (Beijing, China). The lactate dehydrogenase (LDH) assay kit was purchased from Nanjing Jiancheng Bioengineering Institute (Nanjing, China). Hoechst 33258 was obtained from Beijing Solarbio Science & Technology Co. Ltd. (Beijing, China). The enhanced BCA protein assay kit was obtained from Beijing TransGen Biotech Co. Ltd. (Beijing, China).

### 2.2. HPLC Analysis

BSHX extracts were analyzed using a Shimadzu prominence liquid chromatography platform (Kyoto, Japan) equipped with two LC-20AT pumps, CTO-20A column oven, DGU-20A5R degasser, SIL-20A autosampler, and SPD20AD detector. Chromatographic separation was conducted on a AichromBond-AQ C18 column (250 mm × 4.6 mm, 5 *μ*m; Abel Industries Ltd., Canada), protected by a Phenomenex® C18 guard cartridge (3 × 4 mm, 5 *μ*m; Torrance, CA, USA). The mobile phase consisted of ACN (A) and 0.1% aqueous formic acid (B) and was delivered at 1.0 mL/min with the following gradient program: 0–35 min, 0–3%B; 35–100 min, 3–22% B; 100–115 min, 22–35%B; 115–130 min, 35–35%B; 130–140 min, 35–100%B; 140–145 min, 40–100%B; and 145–155 min, 100–100%B. The column was maintained at 40°C. At the end of each run, the delivery of 100% A was performed for another 14 min for system reequilibration. The monitor wavelength was set at 235 nm, 254 nm, and 280 nm, respectively.

### 2.3. Cell Culture

bEnd.3 rat cerebral microvascular endothelial cells were purchased from the Cell Center of the Chinese Academy of Medical Sciences (Beijing, China). Cells were maintained in the endothelial cell medium (ECM) supplemented with 10% (v/v) heat-inactivated fetal bovine serum (FBS) and 1% (v/v) penicillin-streptomycin (PS) in a humidified 5% CO_2_ incubator at 37°C.

### 2.4. Sample Treatment

bEnd.3 cells were divided into the control group, model group and BSHX low-dose group, and medium-dose group and high-dose group. First, cells in all groups were cultured to the logarithmic growth phase, then starved with FBS-free ECM for 24 h. Then cells were replaced with the normal ECM containing 10% FBS for further culture. Afterwards, the cells in the model group were stimulated with 300 *μ*g/mL AGEs for 72 hours; the low-dose group was added with 300 *μ*g/mL AGEs and 20 mg/L BSHX; the medium-dose group was added with 300 *μ*g/mL AGEs and 50 mg/L BSHX; and the high-dose group was treated with 300 *μ*g/mL AGEs and 50 mg/L BSHX for 72 h. The control group was not treated with dosing.

### 2.5. Cell Viability Assay

bEnd.3 cells were seeded into a 96-well plate (3500/well) for 24 h, followed by AGEs (300 *μ*g/mL) and BSHX (20, 50, and 100 mg/L, respectively) treatment as mentioned previously. Cell viability was determined using MTT. In brief, add 200 *μ*L MTT solution (0.5 mg/mL) in each well for 4 h at 37°C. Then replace the original supernatant with 200 *μ*L DMSO and continue to incubate for another 2 h. The optical density (OD) values were measured at 570 nm using a microplate reader (Bio-Rad Laboratories Inc., Hercules, CA, USA). Cell viability (%) = [OD (treatment) − OD (blank)]/[OD (control) − OD (blank)] × 100%.

### 2.6. Lactate Dehydrogenase (LDH) Assay

bEnd.3 cells were exposed to AGEs (300 *μ*g/mL) with or without BSHX (20, 50, and 100 mg/L, respectively) as above. Then, use a commercial kit for the detection of LDH release from the cells, according to the manufacturer's instructions. Absorbance was measured at 450 nm. LDH (U/L) = [OD (treatment) − OD (blank)]/[OD (standard) − OD (blank)] × 0.2 (mmol/L) × 1000.

### 2.7. Hoechst 33258 Staining and Annexin V/PI Staining

Cells were grown on cover glasses (NEST, Wuxi, Jiangsu Province, China) placed at the bottom of a 24-well plate (20,000 cells/well) and subjected to AGEs with or without BSHX as described. After 72 hours, the cell culture medium was removed, and 4% paraformaldehyde (500 *μ*L/well) was added to each well for 20 min, followed by Hoechst 33258 solution (1 *μ*g/ml in PBS) staining in the dark for 30 min at room temperature. Images were captured using a fluorescence microscope (IX73, Olympus, Japan) under an excitation wavelength of 352 nm and an emission wavelength of 461 nm [17, 18].

For Annexin V/PI staining, bEnd.3 cells were treated as showed previously incubation in 6-well plates with an initial density of 2.0 × 10^5^ cells per well. All the operations simply followed the instructions. First, harvested cells with the corresponding supernatants. Then, washed them two times with cold Phosphate Buffered Saline (PBS, Gibco) after centrifugation (500 ×g, 5 min). Next, cells were suspended in binding buffer and stained with Annexin V-FITC (5 *μ*L)/PI (5 *μ*L) mixture for 15 min at room temperature. Detected within an hour with BD FACSCelesta (BD Biosciences, NJ, USA), and data were analyzed using FlowJo software (BD, Version: 10.7.2).

### 2.8. Protein Identification by Nano LC-MS/MS

bEnd.3 were divided into the control group, model group, and BSHX high-dose (100 mg/L) group. First, the cells were cultured in ECM containing AGEs (300 µg/mL) with or without BSHX-contained (100 mg/L) AGEs for 72 h. Then cells were scraped with a spatula and washed by PBS. The cell pellet was extracted with NP40 lysate to obtain total proteins and the protein concentration was determined by the BCA method. After the protein concentration was quantified, SDS-PAGE gel electrophoresis and silver staining were performed. Whole protein bands on SDS–PAGE were excised and digested with trypsin. Afterwards, the obtained peptide mixture was filtered through a 0.22 *μ*m micropore membrane to afford mass spectrometry analysis samples. Extracted peptide samples were analyzed by the nano-liquid chromatography coupled with hybrid linear ion trap-Orbitrap mass spectrometer (nanoLC-LTQ-Orbitrap MS/MS) method. The specific operation steps can refer to the previous research of our research group [19].

### 2.9. Bioinformatics Analysis

Proteins were quantified according to the sum of the signal intensity (the peak area of the precursor) of the first order spectrum of each peptide detected. The proteins with expression difference >5 in the control group and model group, as well as the model group and BSHX group, were selected as differential proteins.

The identified differential proteins were uploaded to the Database for Annotation, Visualization, and Integrated Discovery (DAVID^1^) and Protein Analysis Through Evolutionary Relationships database (PANTHER^2^) databases systems, and the gene ontology (GO) protein classification analysis including the cellular component (CC), the molecular function (MF), and the biological process (BP) was performed.

In addition, for signal pathway enrichment analysis, we introduced the identified differential proteins into Cytoscape software (v.3.5.1) and used the attached ClueGO plug-in to analyze the Kyoto Encyclopedia of Genes and Genomes (KEGG), REACTOME pathway, and Wiki pathway. The species was set as mice. *P* < 0.05 was set as the significance threshold, and the default parameters were used for the rest.

### 2.10. Western Blot Analysis

Cells were vortexed in cold RIPA buffer with protease and phosphatase inhibitor (1x) for 30 min. Nuclear and cytoplasmic proteins were prepared using the Nuclear and Cytoplasmic Protein Extraction Kit (Beyotime) as per the manufacturer's protocol. Total proteins were extracted after centrifugation (12,000 rpm, 10 min, 4°C), and protein concentrations were determined using a BCA Assay Kit (TransGen Biotech). Protein samples from each group were subjected to 10%–15% SDS-PAGE gels system and subsequently transferred to polyvinylidene fluoride (PVDF) membranes (Millipore, Billerica, MA, USA). The membranes were blocked by 5% (w/v) skim milk at room temperature for 60 min, then washed 3 times with Tris-buffered saline containing 0.1% Tween-20 (TBST), incubated with primary antibody (1 : 1000) at 4°C overnight with slight shaking. The membranes were washed 3 times and incubated with secondary antibody (1 : 1000) at room temperature for 2 h. Finally, they were washed another 3 times and exposed to Immobilon Western Chemiluminescent HRP Substrate solution (Millipore). Protein bands were gauged with Tanon 5200 Imaging Analysis System (Tanon Science & Technology Co. Ltd., Shanghai, China). Densitometry analysis of relative protein levels was performed using Gel-Pro analyzer 4 software.

### 2.11. Statistical Analysis

All values were obtained from at least three independent experiments and presented as mean ± S.D. We performed one-way analysis of variance (ANOVA), followed by Bonferroni's post *hoc test*. Differences were considered statistically significant when *P* < 0.05.

## 3. Results

### 3.1. Identification of the Components of BSHX Extract

High-performance liquid chromatography (HPLC) and mass spectrometry (HPLC-MS) analysis were used to identify the ingredients of the BSHX extract. Hundred and fourteen compounds were identified, including 43 flavonoids, 28 small molecular phenolic acids, 17 cinnamoyl polyamines, 11 glycolipids, 8 fatty acids, 4 lignins, 2 semiterpene glycosides, and 1 saponin compound. The HPLC fingerprint of the BSHX extracts and the characterization and sources of these compounds have been reported in our previous article [14].

### 3.2. BSHX Protected bEnd.3 Cells from AGE-Induced Injury

**Figure 1:**
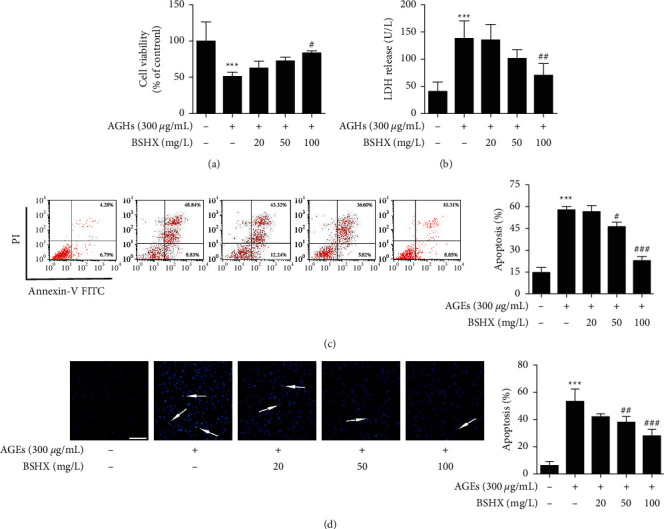
BSHX protected bEnd.3 cells against AGE-induced apoptosis. bEnd.3 cells were exposed to AGEs and treated with BSHX (20, 50, and 100 mg/L, respectively) for 72 h. (a) Cell viability was assessed by MTT and expressed relative to the control group. (b) LDH release was detected with a commercial kit. (c) The effect of BSHX on the AGE-induced apoptosis of bEnd.3 cells was determined by flow cytometry. (d) Apoptotic nuclei were identified using Hoechst 33258 staining. Cells stained by the dye were classified as apoptotic cells (indicated by arrows in the merged images). Scale bar = 100 *μ*m. All data were obtained through at least three independent experiments and presented as mean ± S.D. ^*∗∗∗*^*P* < 0.001, relative to the control group; ^#^*P* < 0.05, ^##^*P* < 0.01, ^###^*P* < 0.001 relative to the model group.

We first used MTT assay to assess the protective effect of BSHX against AGEs. As shown in Figure 1(a), compared with the control group, AGEs (300 *μ*g/mL) led to a significant cell injury (cell viability decreased to 56.55 ± 9.7%, *P* < 0.001), while BSHX treatment (20, 50, and 100 mg/L) restored the cell viability in a dose-dependent manner. Furthermore, we detected the LDH, which released from injured cells, to assess the cell toxicity after drug exposure (Figure 1(b)). We found that LDH released in the supernatant of cells from the model group dramatically swelled to 142.02 ± 13.77 U/L (*P* < 0.001 vs. Control group) and BSHX (100 mg/L) significantly shrunk the release and remedied the cytotoxity (74.83 ± 9.28 U/L, *P* < 0.01 vs. model group). Briefly, our results indicates that BSHX has an excellent protective effect on damaged bEnd.3 cells induced by AGEs.

To further verify the effect of BSHX on cell apoptosis, we stained the cells with Annexin V/PI. Translocation of phosphatidylserine occurs in early apoptotic cells whose cytomembranes are stained by Annexin V only. Incomplete cytomembrane of late apoptotic cells results in nuclei staining by PI. Cells stained by both Annexin V/PI are considered necrotic or fragmented. Dot plots (Figure 1(c)) showed percentage of apoptotic cells (early and late apoptotic cells) in each group—It was 11.07% in the control group, 57.67% in model group, 55.54% in the low dose group (n.s.), 42.42% in medium dose group (*P* < 0.05 vs. model group) and 19.16% in high dose group (*P* < 0.001 vs. model group), respectively.

Moreover, to observe apoptosis directly, cells were subjected to Hoechst 33258 staining which can penetrate the cell membrane and release strong blue fluorescence after embedding in the double stranded DNA of apoptotic cells. The results (Figure 1(d)) revealed that AGEs treatment dramatically promoted chromosome condensation (*P* < 0.001 vs. Control group), which was a crucial indication of cell apoptosis. BSHX of a concentration of 20–100 mg/L effectively attenuated that change (*P* < 0.05 or *P* < 0.001 vs. model group). All of these results suggested that AGEs significantly induced bEnd.3 cell apoptosis and BSHX could protect bEnd.3 cells from AGE insults.

### 3.3. Effects of BSHX on Proteomic Changes Induced by AGEs in bEnd.3 Cells

**Figure 2:**
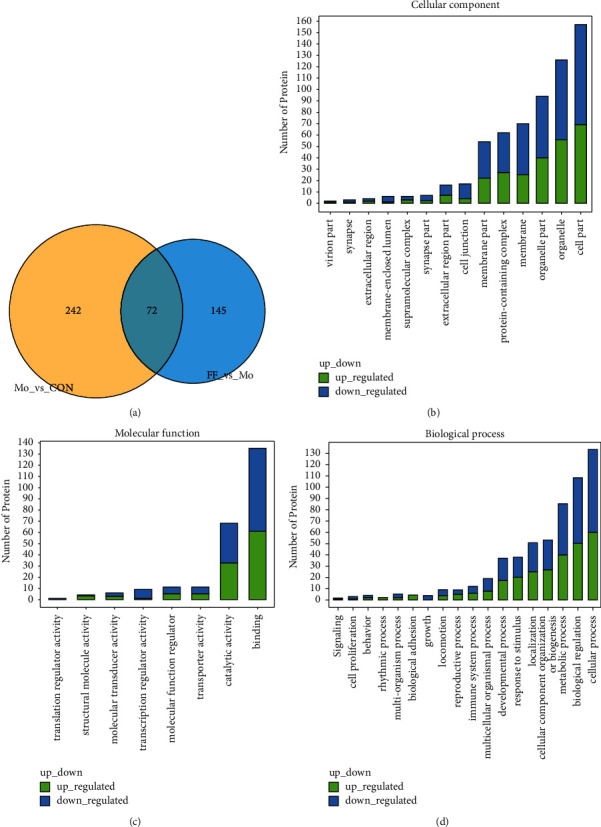
Proteomic analysis results for BSHX-altered proteins. (a) Venn diagram showing numerical distribution of proteins identified in different bEnd.3 cell lysates by the nanoLC-LTQ-Orbitrap MS/MS approach. Cells were treated with vehicle (control group: Con), 300 *μ*g/mL of AGEs (model group: Mo), and AGEs with 100 mg/L of BSHX (high-concentration BSHX treatment group: FF) for 72 h respectively. (b–d) Proteins significantly upregulated and downregulated by BSHX were classified according to the cellular component (b), molecular function (c), and biological process (d).

A quantitative proteomics analysis was carried out using the Nano-LC-MS/MS system to dissect the effect of BSHX on the proteome profile. According to the protein identification results of the bEnd.3 cell lysates based on the Thermo Proteome Discoverer database, there were a total of 314 differentially expressed proteins between the model group and the control group, and 217 proteins were differentially expressed in the BSHX groups and the model group. The relationship of identified proteins in the control, model, and BSHX groups is shown in the Venn diagram (Figure 2(a)).

Comparing the expression levels of the whole proteins in different groups, the differentially expressed protein candidates were acquired. In this study, proteins with a more than fivefold increase or decrease were filtrated. Finally, 314 differentially expressed proteins were identified. Specifically, 223 proteins were upregulated and 91 were downregulated upon AGEs treatment. These 314 differentially expressed proteins were further analyzed between the model and the BSHX groups. Results showed that the expression levels of 107 (47.9%) upregulated proteins and 19 (20.9%) downregulated proteins in the model group were markedly reversed in response to BSHX treatment.

In addition, to explore the impact of BSHX on proteomic changes, the proteins with specific response to BSHX treatment were annotated and categorized according to gene ontology (GO) protein classification analysis (including CC, MF, and BP). As shown in Figure 2(b), these proteins were mainly distributed in organelles or organelle parts (124 downregulated proteins) and the membrane system (77 downregulated proteins). Functionally, they participated in activities as diverse as the binding activity (74 downregulated proteins), the catalytic activity (35 downregulated proteins), and the transcription regulation activity (8 downregulated proteins) (Figure 2(c)). Moreover, these proteins were involved in complicated BPs including cellular processes (73 downregulated proteins), biological regulation (61 downregulated proteins), metabolic processes (45 downregulated proteins), and so on. (Figure 2(d)) Taken together, the above-mentioned results indicated that BSHX notably downregulated the expression of AGE-irritated proteins and effectively blocked the related multiple BPs.

### 3.4. Bioinformatics Analysis for the BSHX-Regulated Signaling Networks

**Figure 3:**
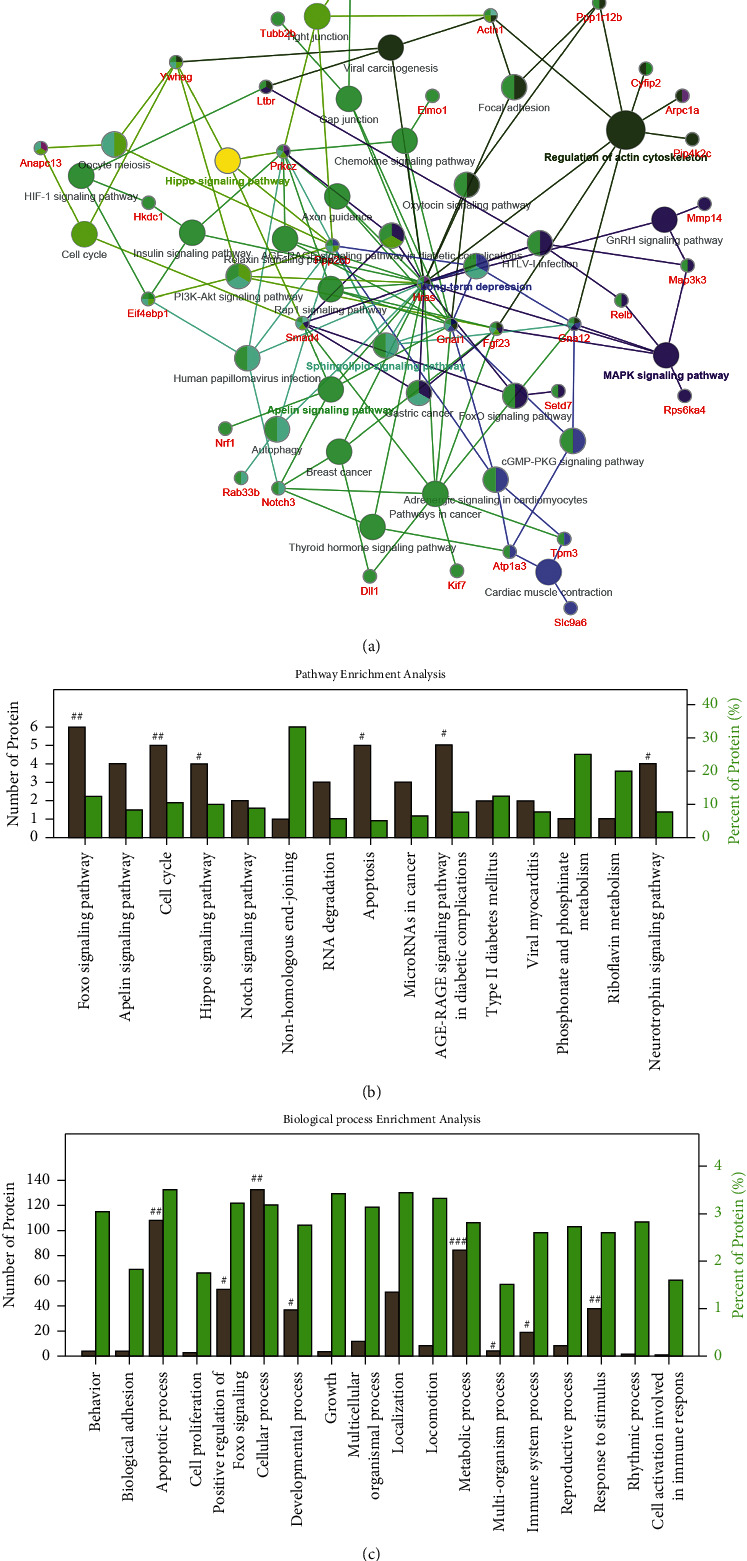
Bioinformatics analysis of the signaling networks negatively regulated by BSHX. (a) Interaction network of protein groups downregulated by BSHX. (b) Pathway enrichment analysis of the differentially expressed proteins predicted the significantly canonical pathways targeted by BSHX in AGE-stimulated bEnd.3 cells. The gray columns on the left *Y*-axis represent the number of identified proteins in each pathway. The green columns on the right *Y*-axis depict the percentage of identified proteins over the total proteins in that pathway. (c) BP enrichment analysis for differentially expressed proteins in BSHX-treated bEnd.3 cells. ^#^*P* < 0.05, ^##^*P* < 0.01, ^###^*P* < 0.001 relative to the model group.

**Figure 4:**
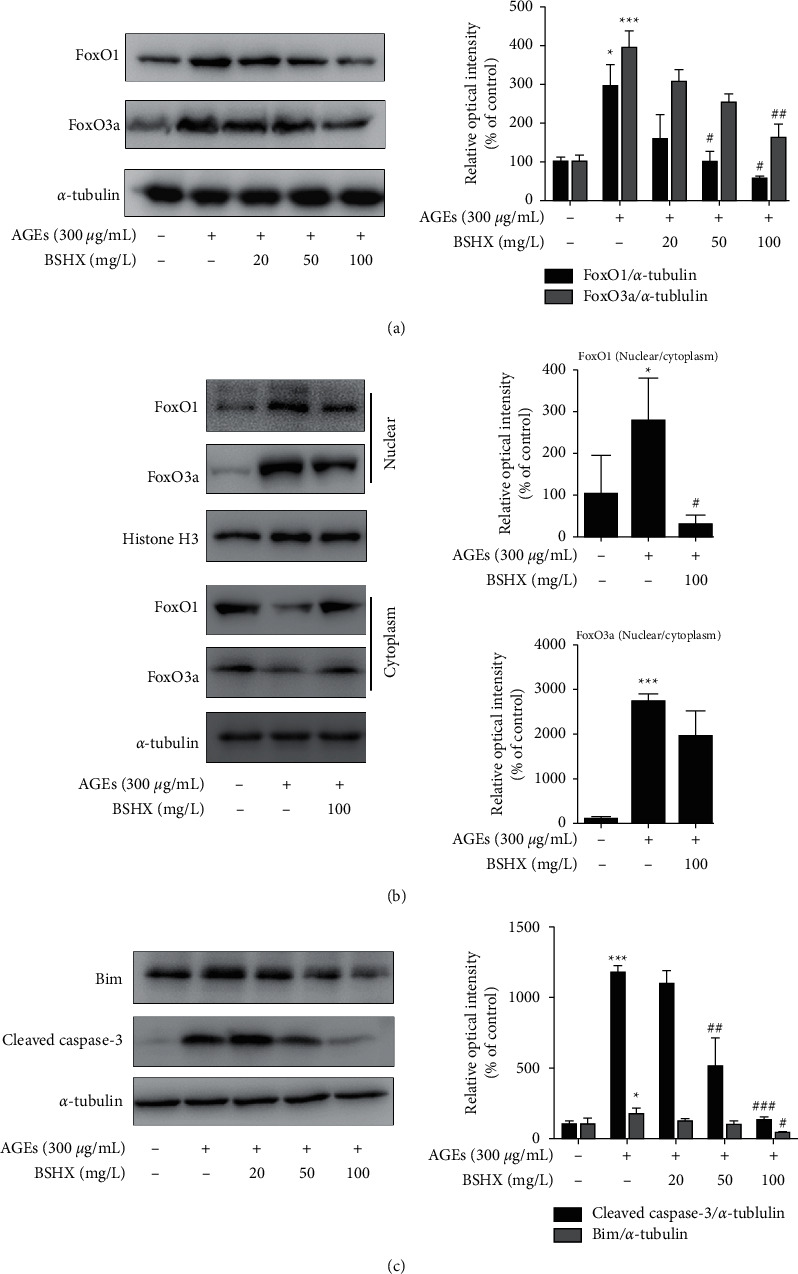
BSHX prevented FoxO1 and FoxO3 proteins from translocating into the nucleus and promoted their degradation under AGE-induced stimulation. (a) bEnd.3 cells were treated with AGEs (300 *μ*g/mL) with or without BSHX (20, 50, and 100 mg/mL, respectively) for 72 h. The total expression of FoxO1 and FoxO3 was determined by Western blot assay. (b) The nuclear and cytoplasmic expression of FoxO1 and FoxO3 in bEnd.3 cells treated with AGEs (300 *μ*g/mL) with or without BSHX (100 mg/mL) for 1 h was determined by Western blot assay. Histone H3 and *α*-tubulin were utilized as internal controls for nuclear and cytoplasmic proteins, respectively. (c) bEnd.3 cells were treated as in (a) for 72 h and then the expression of Bim and cleaved caspase-3 was detected by Western blot assay. All data were acquired by at least three independent experiments and presented as mean ± S.D. ^*∗∗*^*P* < 0.01, ^*∗∗∗*^*P* < 0.001 relative to the control group; ^##^*P* < 0.01, ^###^*P* < 0.001 relative to the model group.

To visualize BSHX-regulated pharmacological networks, the 107 proteins downregulated by BSHX were examined for enrichment in KEGG pathway, REACTOME pathway, and Wiki pathway database from ClueGO plug-in. The pathway networks depicting GO terms with a value of *P* < 0.05 were mapped in Figure 3(a) and listed in Figure 3(b). Results showed that the FoxO pathway, cell cycle pathway, Hippo pathway, apoptosis, and AGE-RAGE signaling pathway in diabetic complications and neurotrophin signaling pathway were the most enriched pathways. To confirm this, GO Biological Process enrichment analysis related to the above-mentioned functionally annotated proteins was utilized. Result showed that multiple biological processes were significantly enriched, including apoptotic process, positive regulation of Foxo signaling, developmental process, metabolic process, response to stimulus, regulation of immune system process, and cell activation involved in immune response (Figure 4(c)). All these biological processes are related to apoptosis. Together, these findings suggested that BSHX could effectively extinguish multiple AGE-activated pathways and BPs and exerted antiapoptotic activity.

### 3.5. BSHX Suppressed FoxO1/3 Pathway in AGE-Induced bEnd.3 Cells

FoxOs transcription factors have been revealed to be closely associated with the occurrence and development of diabetes and its complications [20, 21]. In Figure 4(a), the expression of FoxO1 and FoxO3 proteins was significantly enhanced by AGEs stimulation (*P* < 0.01 or *P* < 0.001), while BSHX markedly restrained the overexpression of these two proteins in a concentration-dependent manner (*P* < 0.01) (Figure 4(a)). To further explore the effect of BSHX on the nuclear translocation of FoxO1/3 that is critical to FoxO1/3-dependent biological processes, the expression of FoxO1 and FoxO3a in the nucleus and cytoplasm of bEnd.3 cells treated with AGEs and a high dose of BSHX (100 mg/L) for 1 h were detected. Results suggested that the expression of FoxO1 and FoxO3a in the nucleus of AGE-challenged bEnd.3 cells was sharply increased (*P* < 0.001), while their expression in the cytoplasm of such cells was decreased (Figure 4(b)). However, 100 mg/L BSHX markedly blocked the nuclear translocation of FoxO1 and FoxO3a (*P* < 0.001) as demonstrated by the reduced FoxO1/FoxO3a levels in the nucleus and the elevated FoxO1/FoxO3a levels in the cytoplasm (Figure 4(b)). These findings implied that BSHX could inhibit the AGE-induced nuclear transfer of FoxO1/3.

In addition, to further understand the activation level of the FoxO signaling pathway, we also detected the protein expression levels of Bim and caspase-3 as well as the downstream target gene of FoxO transcription factors. It can be seen from Figure 4(c) that after stimulating the cells with AGEs for 72 h, the protein levels of Bim and activated caspase-3 were increased significantly (*P* < 0.001), while BSHX reduced their expression in a dose-dependent manner (*P* < 0.001). These findings indicated that BSHX inhibited the expression and AGE-induced nuclear transfer of FoxO1/3 and downregulated the expression of apoptosis-related proteins Bim and caspase-3, thus arresting the activation of the FoxO pathway and protecting cells from apoptosis.

## 4. Discussion

Cognitive dysfunction is increasingly recognized as one of the most severe complications of diabetes mellitus, which can be manifested as short-term memory and executive function impairment. It has also been acknowledged that diabetes is one of the independent risk factors for cognitive dysfunction. As established by pathological studies, similar to patients with Alzheimer's disease (AD), the brains of diabetes-associated cognitive dysfunction patients are also featured by amyloid deposition and abnormal phosphorylation of tau proteins [22]. What's more, this complication and AD share many common mechanisms of pathogenesis, such as insulin resistance, cytotoxicity of AGEs, oxidative stress, and inflammation. Therefore, scholars have recently presented a hypothesis that AD is a kind of type 3 diabetes [23–25].

In our experiments, we studied AGEs, a common pathogenic factor of diabetic cognitive dysfunction and AD. AGEs have cytotoxicity and are a major product of nonenzymatic catalysis reactions of the aldehyde group of glucose and the amino group of proteins. During the normal aging process, AGEs may exist in different cells, but the AGEs levels in patients with diabetes and AD are significantly increased. In patients with T2DM, hyperglycemia accelerates the accumulation of AGEs in kidneys, retinal vessels, and the central nervous system [26–28]. The combining of AGEs with their receptor RAGE can activate a variety of proinflammatory cytokines and produce a large number of reactive oxygen species (ROS), resulting in mitochondrial dysfunction and cell apoptosis. Recent studies found that the increase of AGEs could cause the hyperphosphorylation of tau proteins and the accumulation of A*β*, thereby accelerating the progress of cognitive impairment and neurodegeneration [29].

BSHX prescription is to add leeches on the basis of the Wuzi Yanzong prescription (*Icariside*, *Cuscuta chinensis*, *Chinese wolfberry*, *Rubus idaeus*, *Schisandra chinensis*, *plantaginis semen*). Leeches have blood stasis removing and menstrual flow restoring effects. BSHX can activate blood circulation to dredge collaterals and tonify the kidney. The clinical application has validated its effectiveness in alleviating memory decline and reducing blood sugars. Apoptosis is a process of programmed cell death, which plays a crucial role in cell death and has been widely studied for neuroprotective agent development [30]. In this study, the neuroprotective effect of BSHX on AGE-induced cytotoxicity and the related potential mechanism were analyzed. The results showed that AGEs insults remarkably reduced the viability of bEnd.3 cells, while BSHX tremendously improved the cell survival rate and inhibited LDH release in a concentration-dependent manner, confirming the neuroprotective effect of BSHX. In addition, Hoechst 33258 and Annexin V/PI staining results demonstrated that AGEs stimulation induced the fragmented or shrunken nuclei, which, however, were effectively inhibited by BSHX. All in all, BSHX could inhibit bEnd.3 cell apoptosis.

Proteomics is a discipline that studies the qualitative, quantitative, and modified status of all proteins in complete cells, tissues, body fluids, and other samples based on mass spectrometry technology [31]. The research ideas of proteomics are very similar to the holistic and multitarget views of traditional Chinese medicine (TCM). In the study of pharmacology of TCM, proteomics technology can be used to explore the change of protein level of TCM itself. At the same time, we can also perform high-throughput detection of protein expression levels before and after medication, compare the differences in protein expression of cell or tissue samples under different physiological or pathological conditions, and identify and quantify the related proteins, so as to analyze the interactions between proteins and biological functions at a global level. At present, proteomics has successfully revealed the targets and pharmacological mechanisms of many active molecules of traditional Chinese medicine, such as arsenictrioxide, ganoderic acid, and gambogic acid [32–34]. In this study, we applied the integrated proteomics and bioinformatics to identify proteins regulated by BSHX and their biological functions. The LC-MS/MS-based shotgun proteomics analysis showed that BSHX negatively regulated multiple AGE-elicited proteins. Bioinformatics analysis revealed that these differential proteins were involved in multiple processes including FoxO pathway, apoptosis, AGE-RAGE signaling pathway in diabetic complications and so on. In addition, multiple Biological Processes, such as apoptotic process, positive regulation of Foxo signaling, metabolic process, regulation of immune system process were significantly enriched. All these findings suggested that BSHX could effectively extinguish multiple AGE-activated pathways and BPs, and exerted antiapoptotic activity.

The FoxO signaling pathway is closely related to the occurrence and development of diabetes and its complications [35, 36], and involved in glucose metabolism, apoptosis, inflammation and other biological processes. Exposed to oxidative stress and other harmful stimuli, FoxO proteins in the cytoplasm are dephosphorylated and transferred to the nucleus, where they bind to DNA and promote the expression of downstream apoptotic genes, such as Bim and Fas ligands, consequently initiating the apoptosis [37–39]. Based on this standpoint, the FoxO pathway was studied in this paper, and two most well-understood FoxO proteins, FoxO1 and FoxO3, were further examined to ascertain the detailed mechanism. In line with the proteomic analysis results, Western blot experiments corroborated the inhibitory effects of BSHX on the AGE-activated FoxO pathway. BSHX was shown to regulate the subcellular localization of FoxO1 and FoxO3 proteins, downregulate their protein levels, inhibit their nuclear transfer, curb the expression of downstream apoptotic protein Bim, and prevent the activation of caspase. In this regard, our work demonstrated the beneficial role of BSHX in the regulation of the FoxO pathway, and the effectiveness of direct pharmacological inactivation of FoxO1/3 in treating brain microvascular damage in T2DM patients.

In addition, it is noteworthy that there might be some differences between the bEnd.3 cell line and the primary cells. Although we have identified the BSHX-regulated anticerebral microvascular endothelial injury signaling networks in bEnd.3 cells, a further validation for these above-mentioned crossed signaling pathways in primary cells is warranted and the direct molecular target of BSHX requires identification in future studies.

## 5. Conclusions

In summary, the current study demonstrated the neurovascular protective effect of BSHX on a classical AGE-stimulated bEnd.3 cell model and highlighted the related mechanism by an integrated approach of label-free quantitative proteomics and molecular biology analysis. BSHX, a traditional Chinese medicine, suppressed multiple AGE-activated pathways, such as FoxO/Bim/Caspase-3 signaling pathway to interrupt the expression of various apoptotic proteins, thereby preventing brain microvascular endothelial cells from apoptosis. The study findings suggest the potential therapeutic targets for BSHX in the management of diabetic cerebral microangiopathy and offer insights into the proteomics-guided pharmacological mechanism study of Traditional Chinese Medicine.

## Figures and Tables

**Table 1:** Composition of BSHX.

Herbal composition	Chinese name	Part used	Percentage of total weight
*Cuscuta chinensis* Lam.	Tu Si Zi	Fruit	25
*Lycium barbarum* L	Gou Qi Zi	Fruit	25
*Rubus chingii* Hu.	Fu Pen Zi	Fruit	12
*Schizandra chinensis (Turcz.)* Baill.	Wu Wei Zi	Fruit	3
*Plantago asiatica* L.	Che Qian Zi	Seed	6
*Epimedium brevicornu* Maxim.	Yin Yang Huo	Stem Leaf	25
*Hirudo nipponica* Whitman	Shui Zhi	The whole body	4

## Data Availability

The datasets used and analyzed during the current study are available from the corresponding author on reasonable request.

## Abbreviations

A*β*: Amyloid-*β* protein
AD: Alzheimer's disease
AGEs: Advanced glycosylation end products
BP: The biological process
BSHX: Bushen Huoxue prescription
CC: The cellular component
DM: Diabetes mellitus
DMEM: Dulbecco's modified eagle's medium
ECM: The endothelial cell medium
FBS: Fetal bovine serum
KEGG: The Kyoto Encyclopedia of Genes and Genomes
LDH: The lactate dehydrogenase
MF: The molecular function
RAGE: Receptors of AGEs
ROS: Reactive oxygen species
T2DM: Type 2 diabetes mellitus.

## Supplementary Materials

Supplementary Materials Supplementary file includes the original data of Figure 4.
