# Supplementary figures and images for: A Study of the Protective Effect of Bushen Huoxue Prescription on Cerebral Microvascular Endothelia Based on Proteomics and Bioinformatics
Source: Evid Based Complement Alternat Med. 2022 Jan 6;2022:2545074. doi: 10.1155/2022/2545074 (PMC8758271; doi:10.1155/2022/2545074)

**Supplementary file**

The Original Data of Figure 4


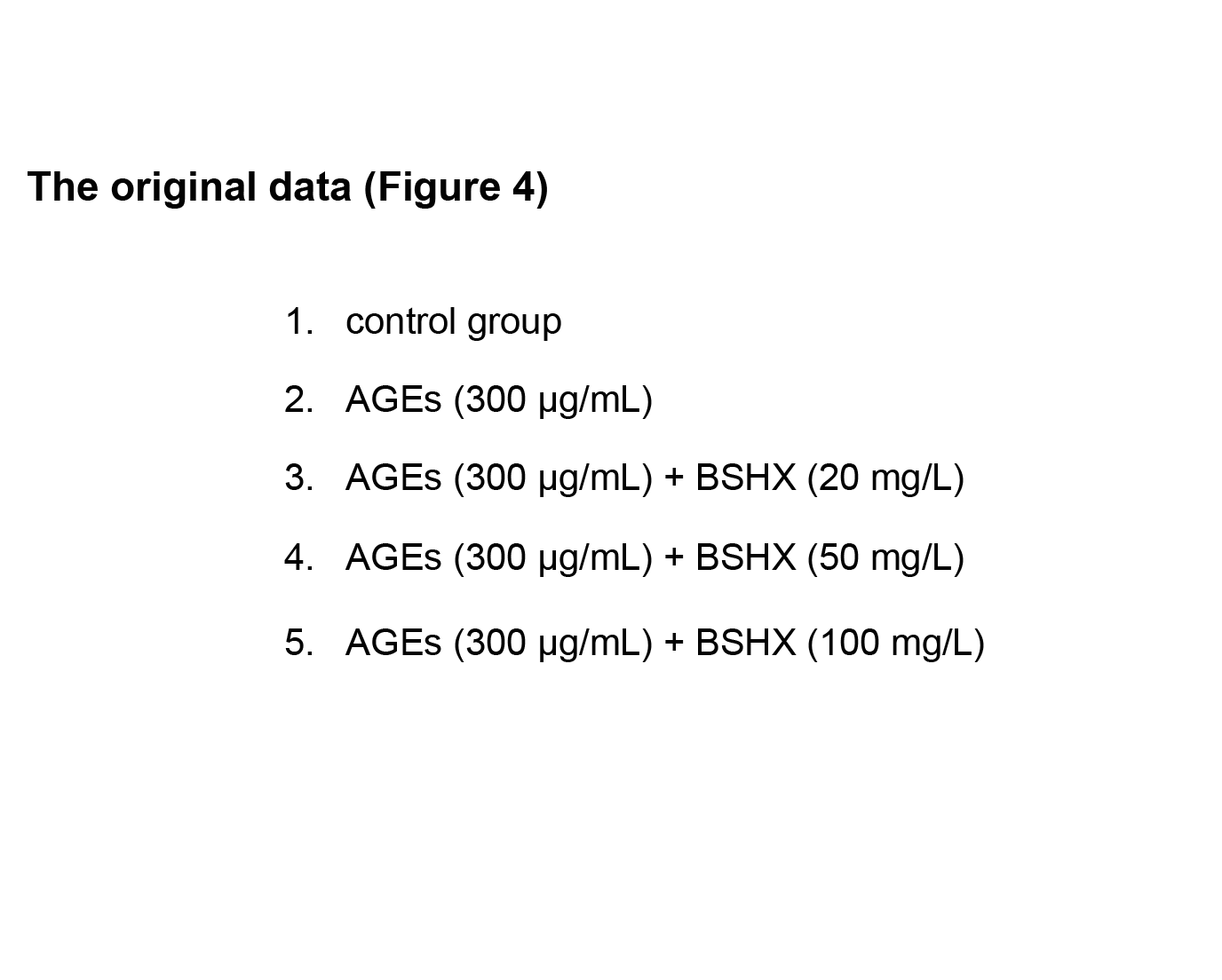


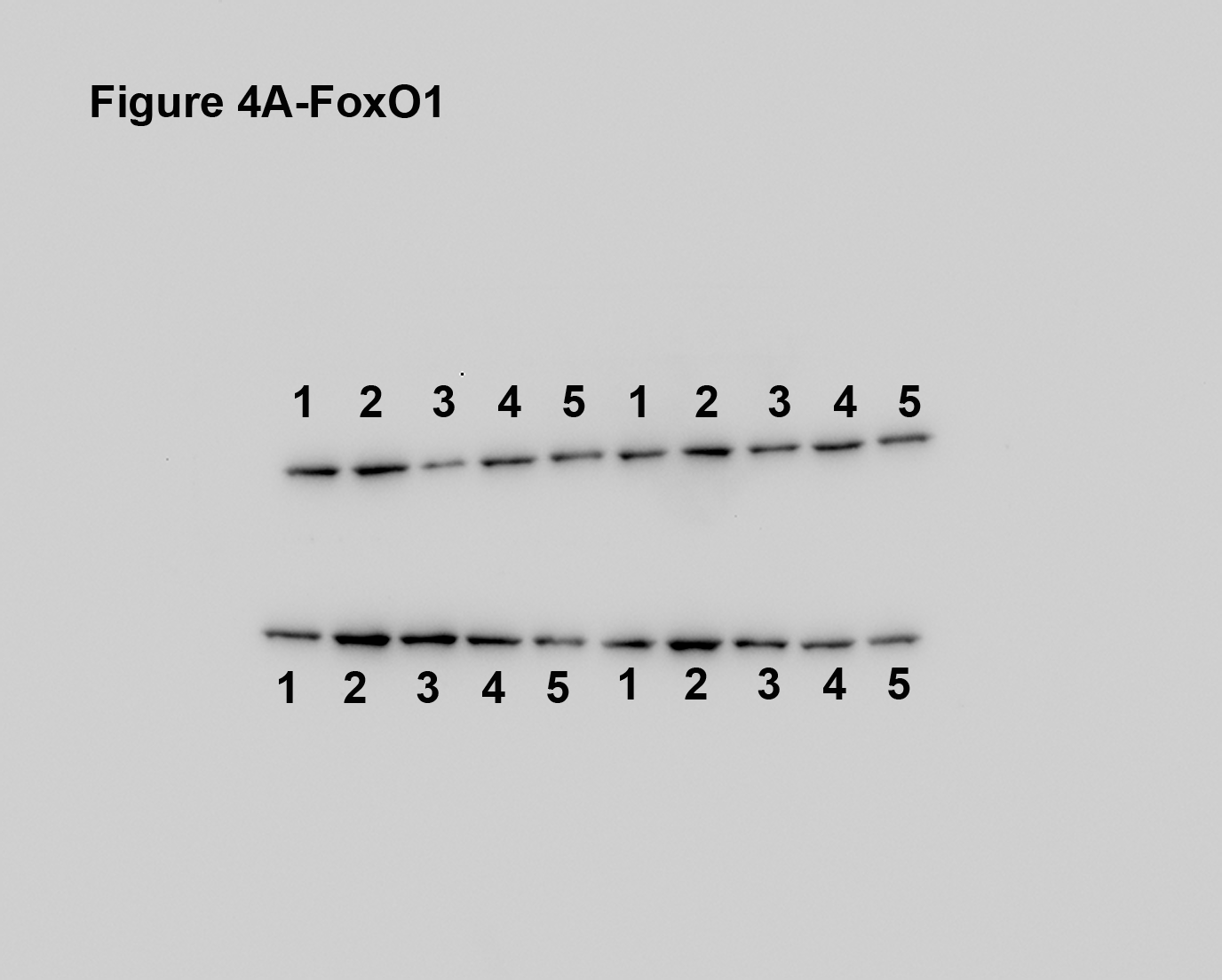


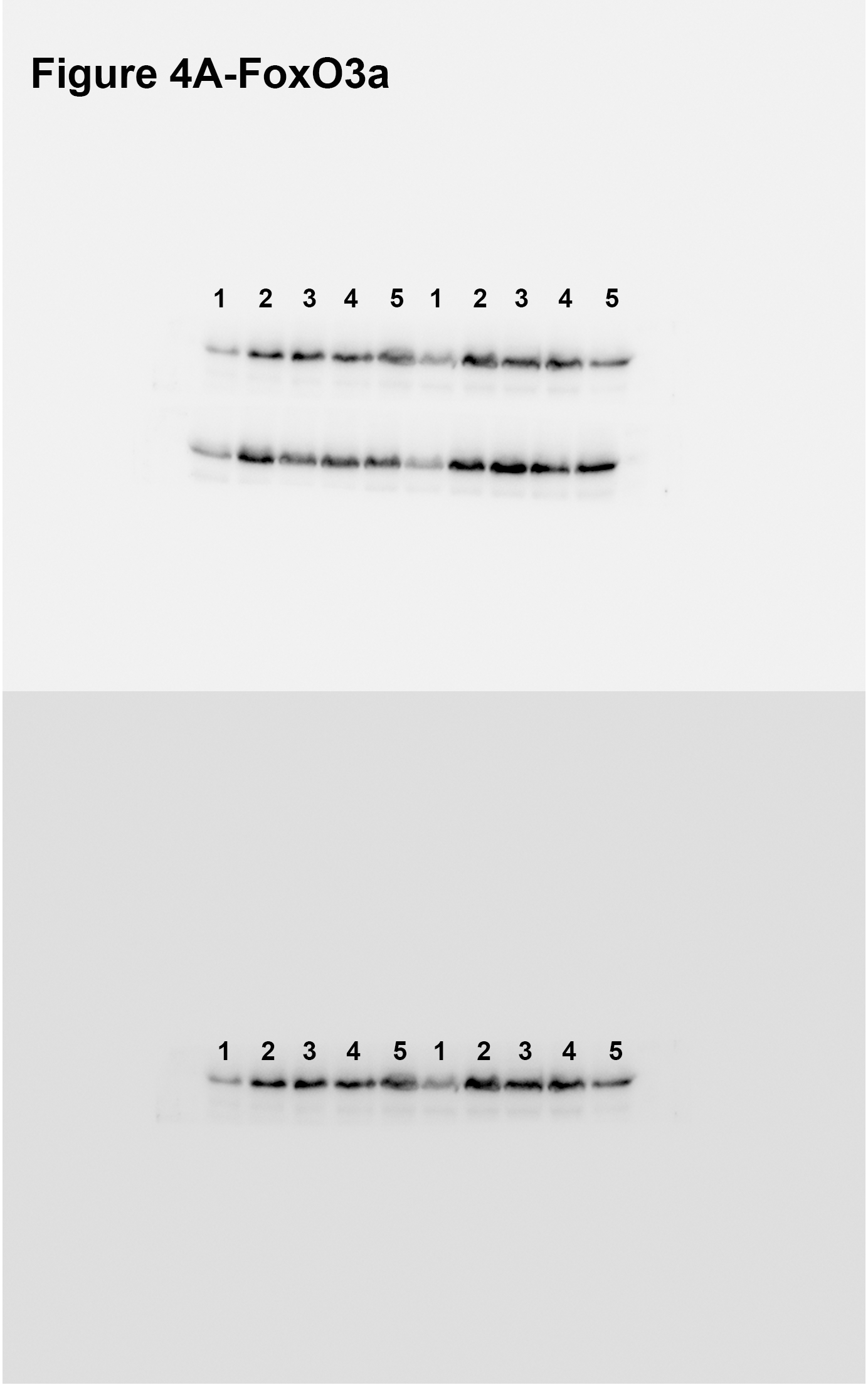


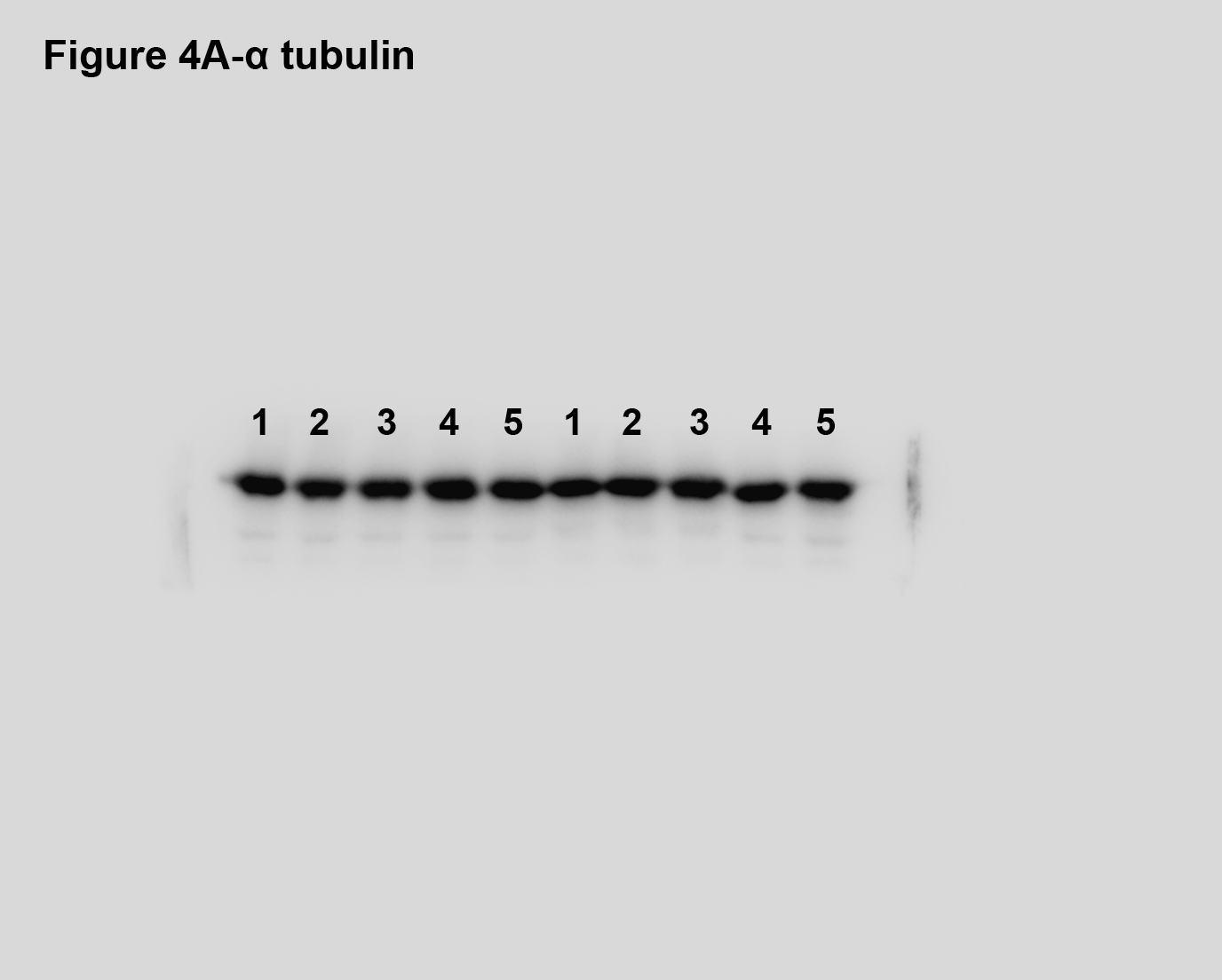


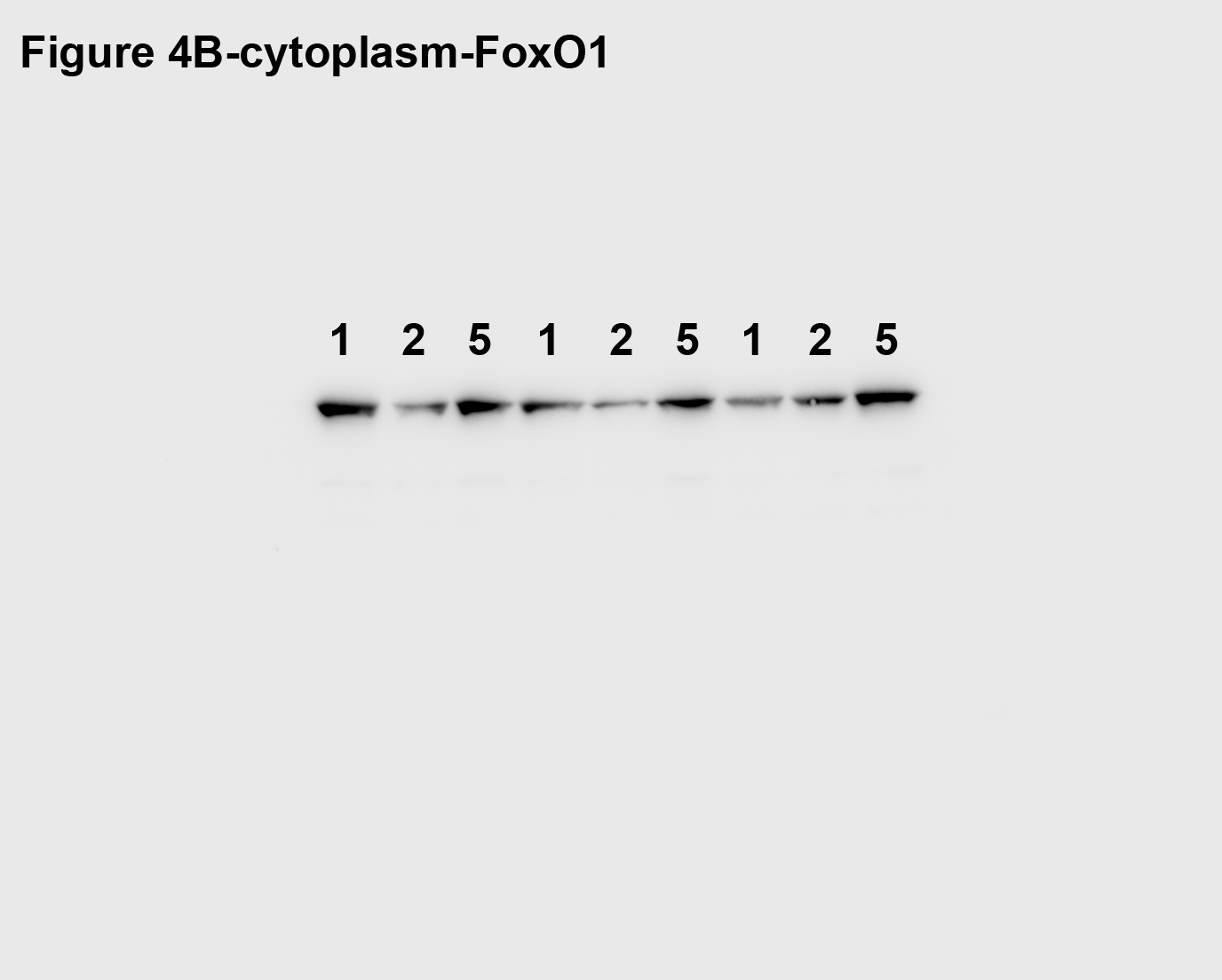


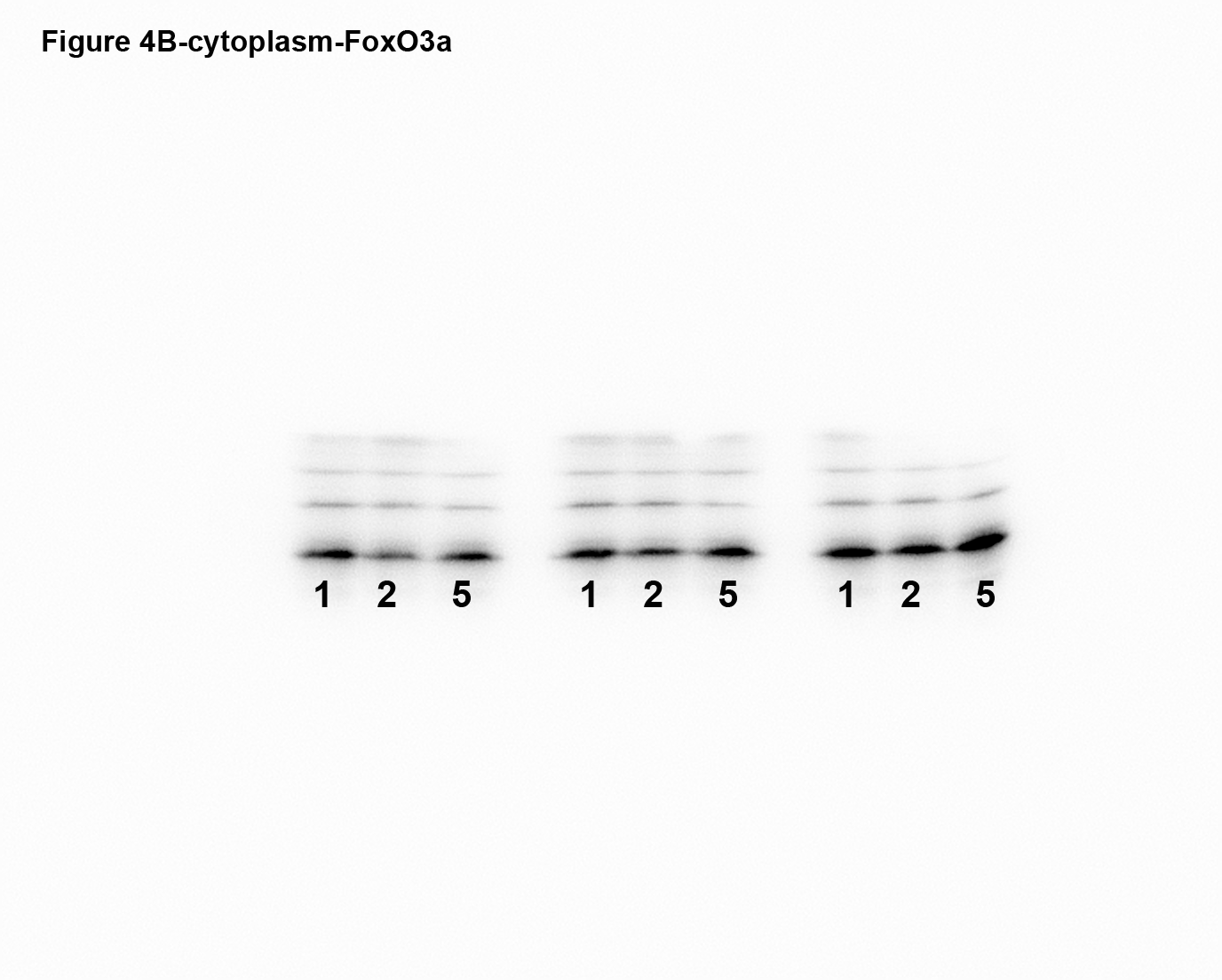


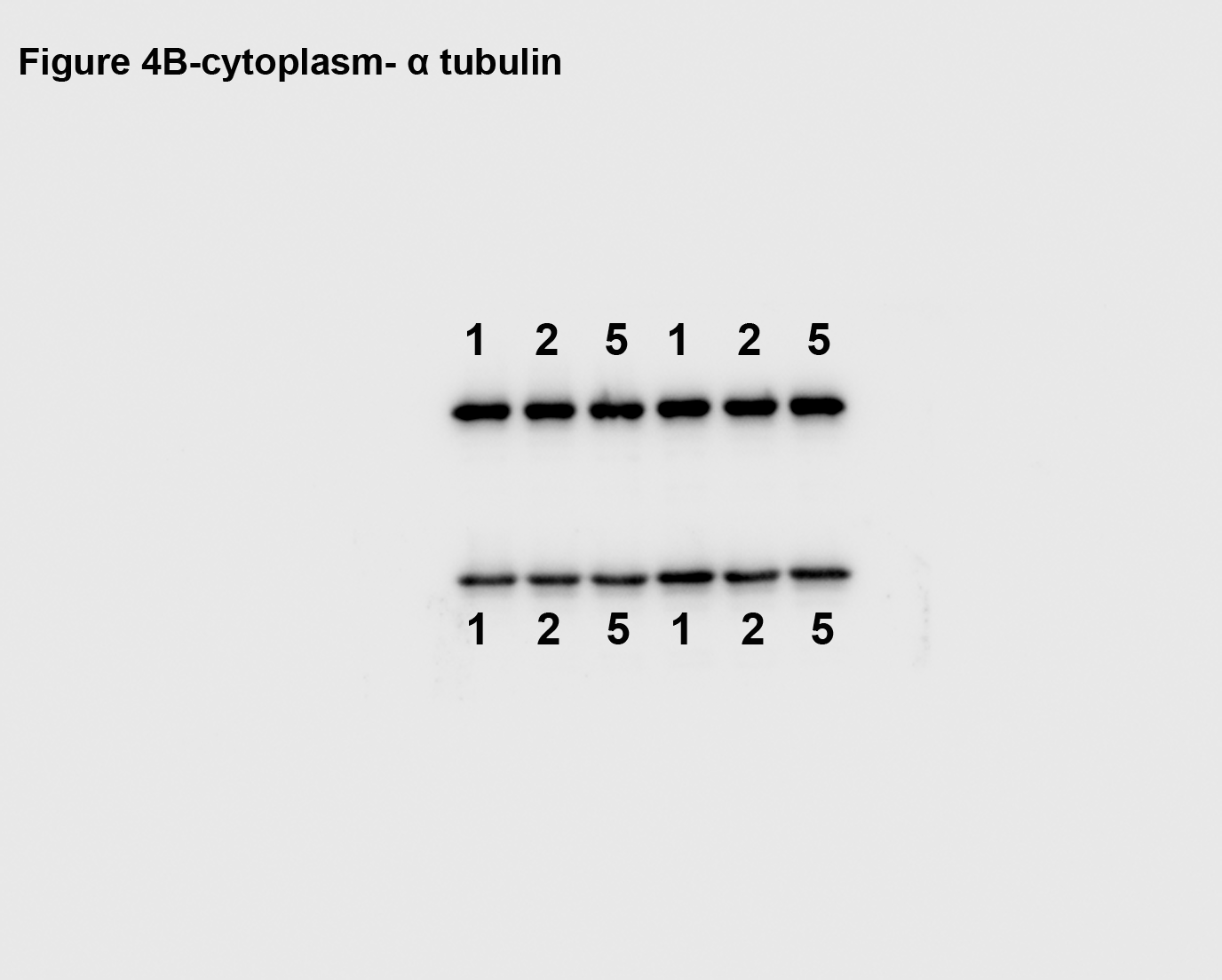


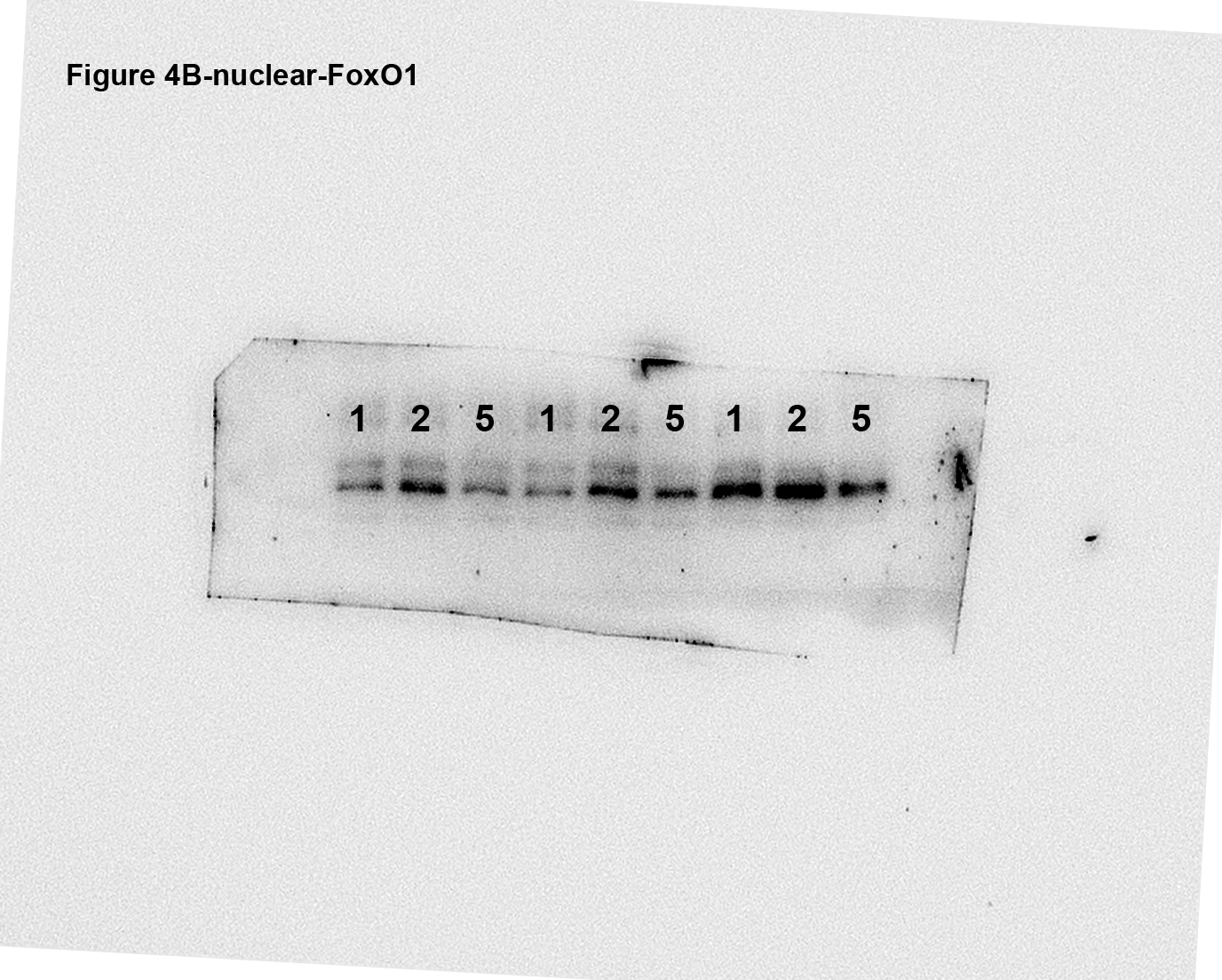


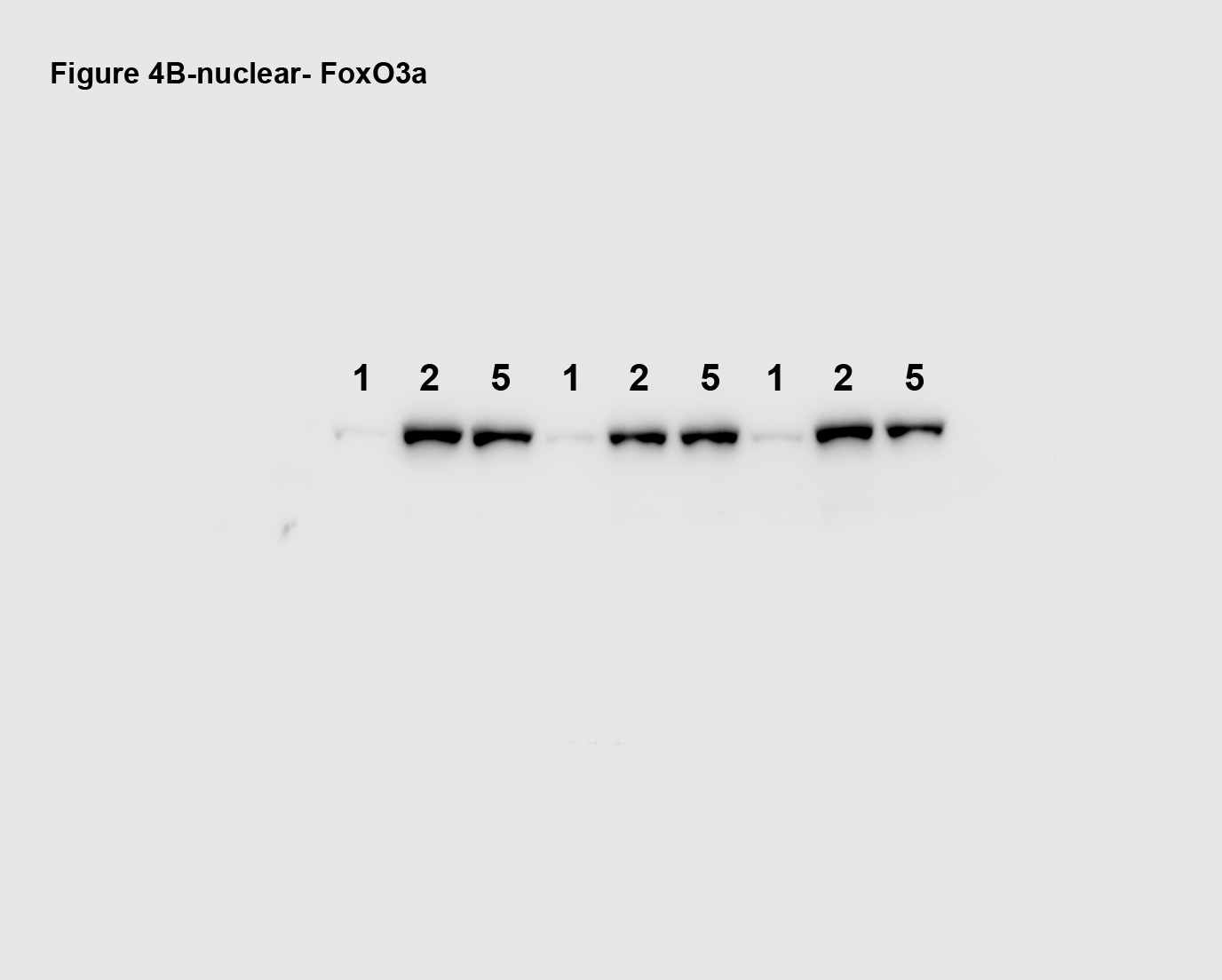


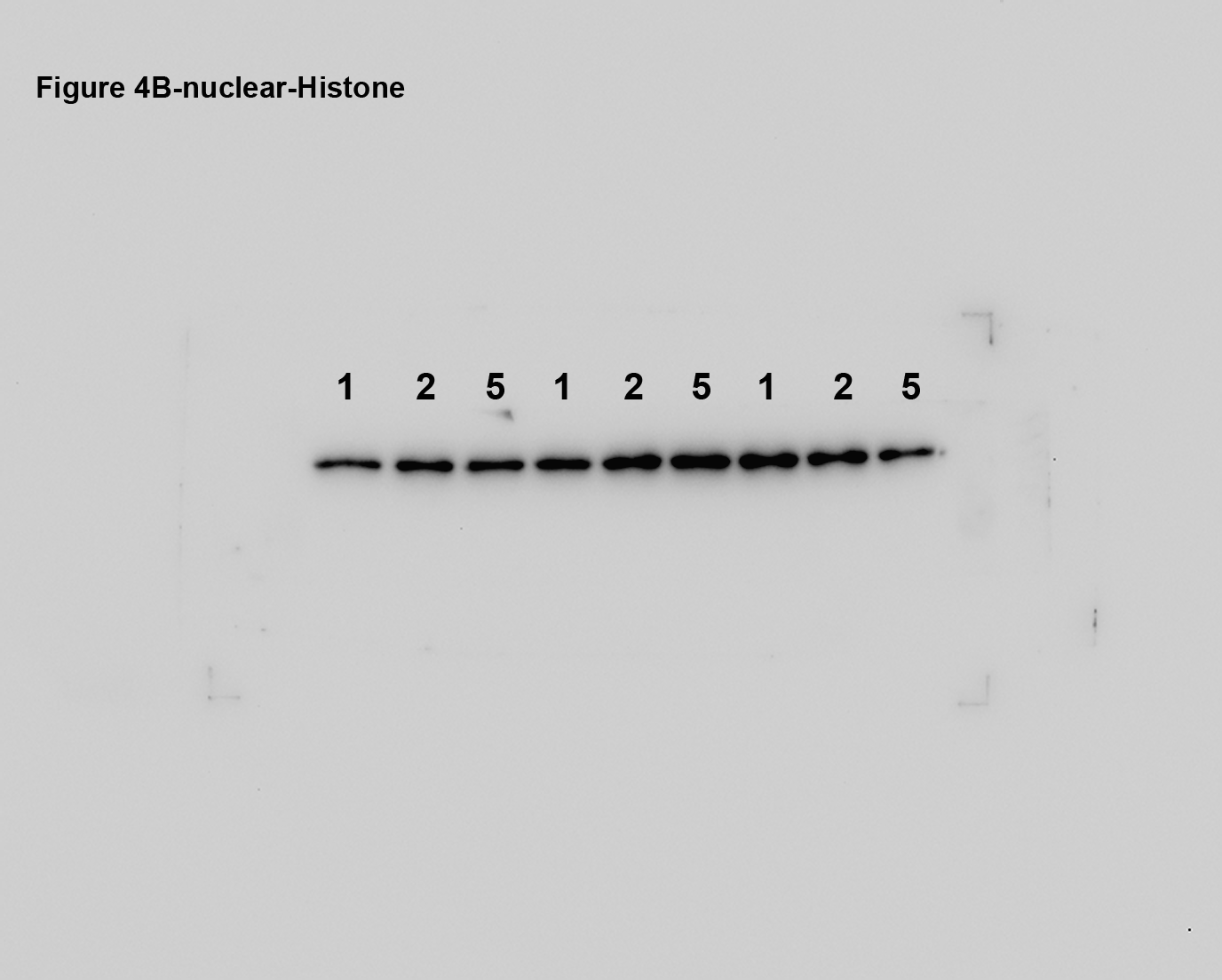


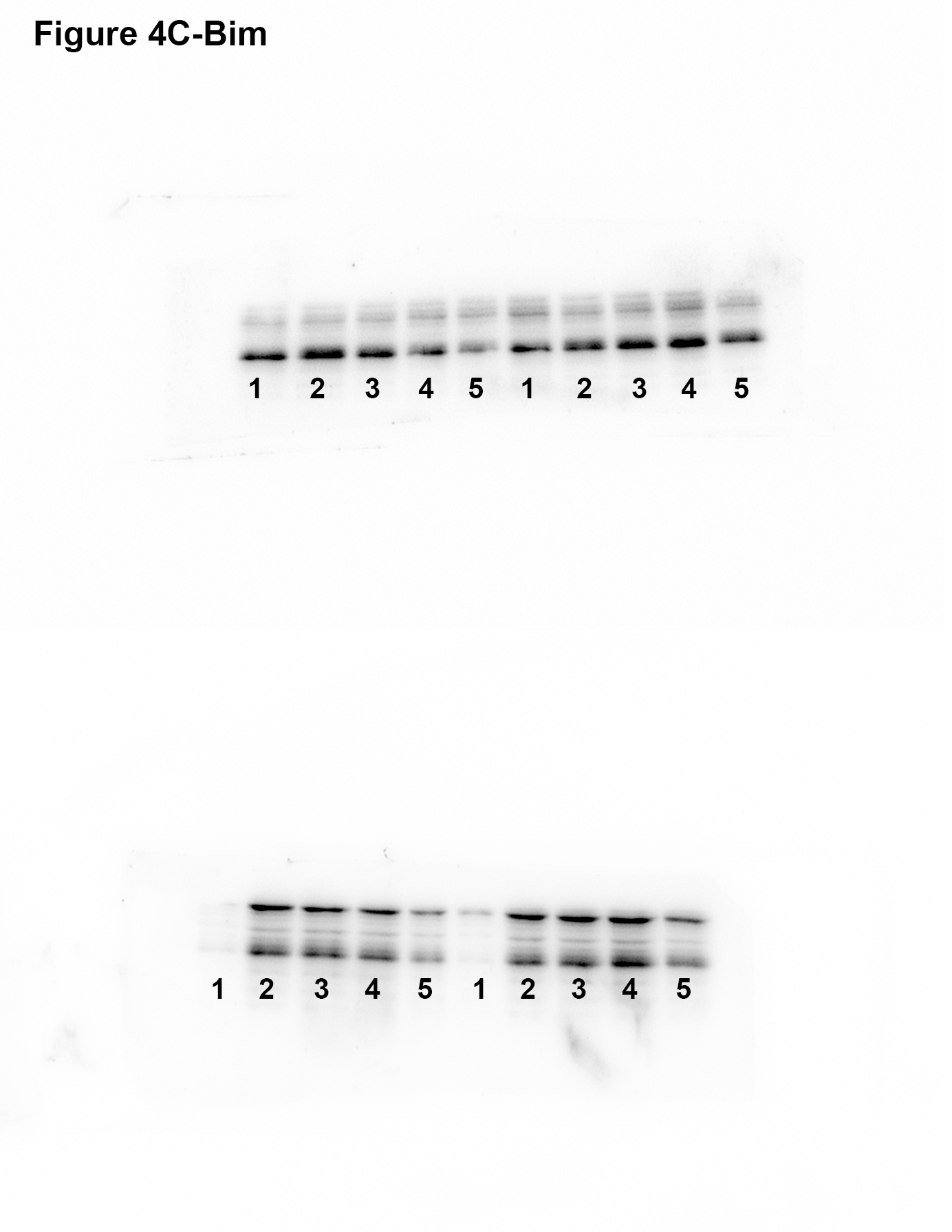


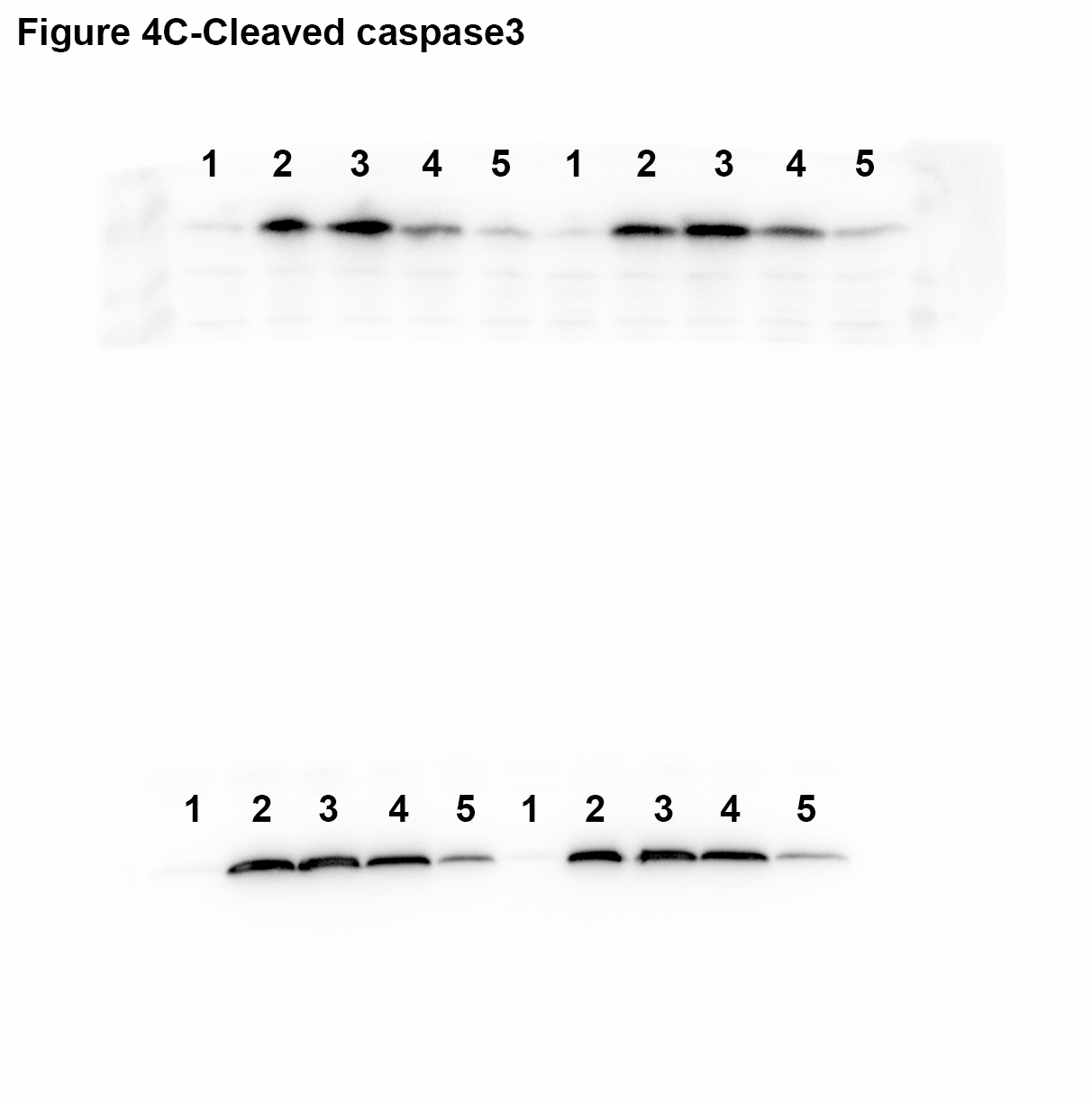


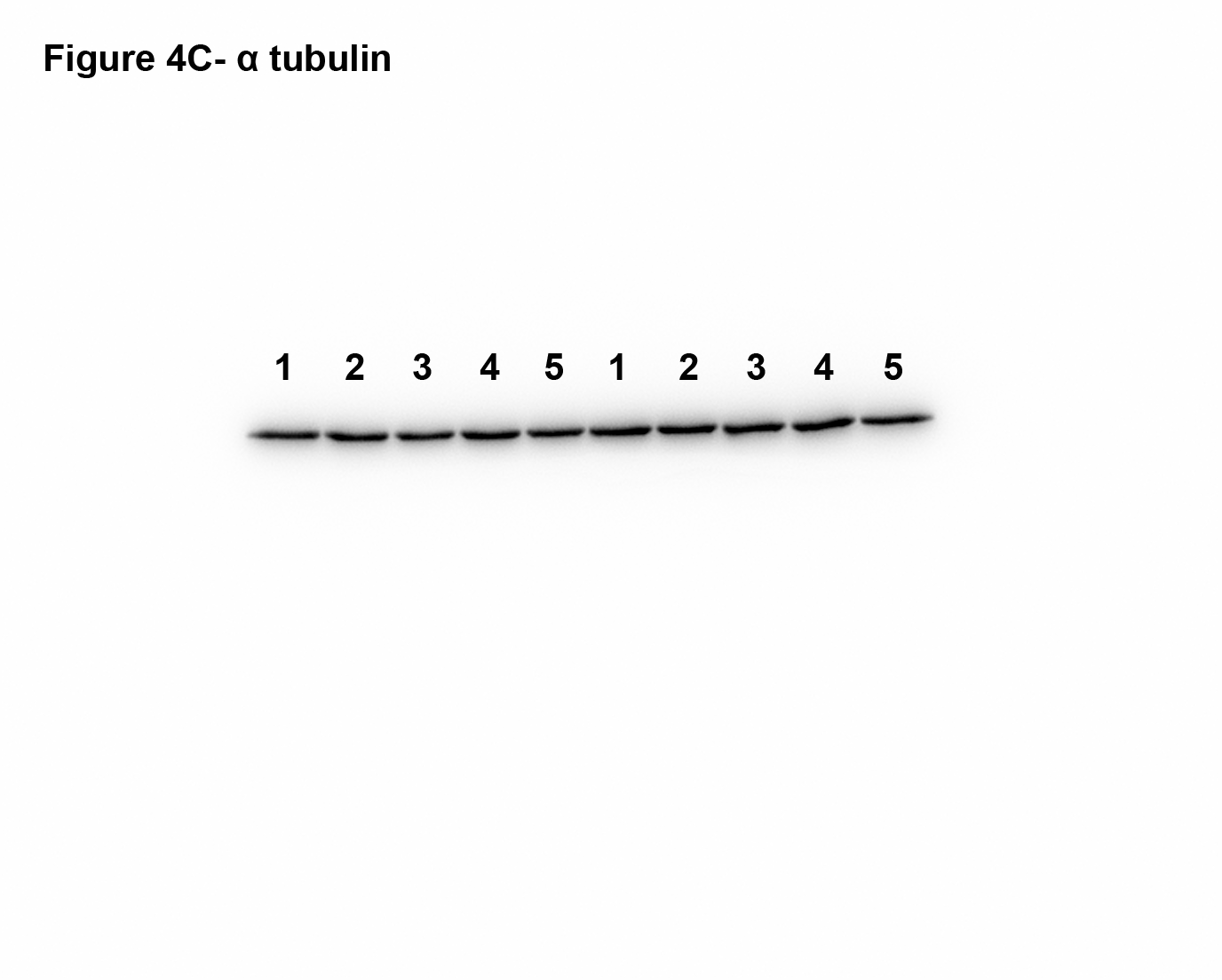

Supplement: Supplementary Materials — Supplementary file includes the original data of Figure 4. [file 2545074.f1.doc]
